# Health in conflict and post-conflict settings: reproductive, maternal and child health in Colombia

**DOI:** 10.1186/s13031-020-00273-1

**Published:** 2020-05-27

**Authors:** Sara Milena Ramos Jaraba, Natalia Quiceno Toro, María Ochoa Sierra, Laura Ruiz Sánchez, Marlly Andrea García Jiménez, Mary Y. Salazar-Barrientos, Edison Bedoya Bedoya, Gladis Adriana Vélez Álvarez, Ana Langer, Jewel Gausman, Isabel C. Garcés-Palacio

**Affiliations:** 1 Grupo de Epidemiologia, Facultad Nacional de Salud Pública, Universidad de Antioquia UdeA, Calle 70 No. 52-72, Medellín, Colombia; 2 Grupo Cultura, Violencia y Territorio, Instituto de Estudios Regionales, Universidad de Antioquia UdeA, Calle 70 No. 52-72, Medellín, Colombia; 3 Hegemonía, Guerras y Conflictos, Instituto de Estudios Políticos, Universidad de Antioquia UdeA, Calle 70 No. 52-72, Medellín, Colombia; 4 Facultad de ciencias sociales y humanas, Universidad de Antioquia UdeA, Calle 70 No. 52-72, Medellín, Colombia; 5 Nacer Salud Sexual Reproductiva, Facultad de Medicina, Universidad de Antioquia UdeA, Calle 70 No. 52-72, Medellín, Colombia; 6 Facultad Nacional de Salud Pública, Universidad de Antioquia UdeA, Calle 70 No. 52-72, Medellín, Colombia; 7 Department of Global Health and Population, Harvard T.H. Chan School of Public Health, 651 Huntington Avenue, FXB Building 7th Floor, Boston, USA

## Abstract

**Background:**

In conflict-afflicted areas, pregnant women and newborns often have higher rates of adverse health outcomes.

**Objective:**

To describe maternal and child health indicators and interventions between 1998 and 2016 comparing high and low conflict areas in Colombia.

**Methods:**

Mixed study of convergent triangulation. In the quantitative component, 16 indicators were calculated using official, secondary data sources. The victimization rate resulting from armed conflict was calculated by municipality and grouped into quintiles. In the qualitative component, a comparative case study was carried out in two municipalities of Antioquia: one with high rates of armed conflict and another with low rates. A total of 41 interviews and 8 focus groups were held with local and national government officials, health professionals, community informants, UN agencies and NGOs.

**Results:**

All of the indicators show improvement, however, four show statistically significant differences between municipalities with high victimization rates versus low ones. The maternal mortality ratio was higher in the municipalities with greater victimization in the periods 1998–2004, 2005–2011 and 2012–2016. The percentage of cesarean births and women who received four or more antenatal visits was lower among women who experienced the highest levels of victimization for the period 1998–2000, while the fertility rate for women between 15 and 19 years was higher in these municipalities between 2012 and 2016. In the context of the armed conflict in Colombia, maternal and child health was affected by the limited availability of interventions given the lack of human resources in health, supplies, geographical access difficulties and insecurity. The national government was the one that mostly provided the programs, with difficulties in continuity and quality. UN Agencies and NGOs accessed more easily remote and intense armed conflict areas. Few specific health interventions were identified in the post-conflict context.

**Conclusions:**

In Colombia, maternal and child health indicators have improved since the conflict, however a pattern of inequality is observed in the municipalities most affected by the armed conflict.

## Introduction

Colombia is situated in northwestern South America, and its history during the last 70 years has been marked by internal armed conflict [1]. This conflict has been complicated to understand, not only because it is one of the most prolonged armed conflicts in the world, but also because it extends across the country, involves different legal and illegal armed groups, has distinctive urban and rural characteristics, and is intertwined with other types of violence [2]. Violence perpetrated by armed groups (guerrillas, paramilitaries, sectors of the public force), organized criminal groups (drug traffickers, gangs), as well as common criminal groups often overlap, which can affect the characteristics of the armed conflict itself [3]. While the Colombian armed conflict has been characterized by having some criminal elements, it still remains mostly political in character [4]. The National Center for Historic Memory has divided the armed conflict in Colombia into five periods in order to describe both its territorial and temporal diversity (Table 1) [3, 5].

**Table 1:** History of conflict in Colombia

Period/years	Description	Groups involved
**FIRST PERIOD (1958–1982)**	Emergence of guerrilla forces, subversive violence, and violence between two political parties.	Guerrilla groups: FARC, ELN, M-19, EPL, *Movimiento Armado Quintín Lame, Corriente de Renovación Socialista*, PRT. Colombian Liberal Party, Colombian Conservative Party
**SECOND PERIOD (1982–1996)**	Was defined by the military strengthening of guerrilla forces and their territorial expansion, as well as the appearance of paramilitaries and proliferation of drug trafficking. Also there were peace process with some guerrilla groups.	FARC, ELN Demobilization of some guerrilla groups (EPL and M-19 among others). Paramilitary groups: ACDEGAM, *Autodefensas de Puerto Boyacá, Autodefensas Campesinas del Magdalena Medio*, and ACCU. In 1997 they unified as AUC.
**THIRD PERIOD (1996–2005)**	Exhibited the greatest upsurge in the conflict with the expansion of illegal armed groups, massacres, extortion, kidnapping, drug trafficking.	AUC FARC ELN
**FOURTH PERIOD (2005–2012)**	Known as the period of “readjustment of the armed conflict,” was defined by two crucial processes: 1) a military offensive by the Colombian state, and 2) negotiations and subsequent demobilization of paramilitary groups.	Demobilization AUC FARC ELN
**FIFTH PERIOD (2012–2017)**	Peace dialogues in Havana between the national government and FARC-EP guerillas, dialogue with ELN guerillas in Quito, reconfiguration of other armed groups in the country.	FARC ELN

Fuente: *GMH. ¡BASTA YA! Colombia: Memorias de guerra y dignidad. Bogotá: Imprenta Nacional, 2013*

Illegal armed actors, such as the FARC-EP guerillas, the ELN and the auto-defense and paramilitary groups, used several different types of violence defined as war crimes and crimes against humanity during the course of the conflict [6]; their crimes included kidnapping, extortion, sexual violence, massacres, recruitment of minors and terrorist attacks [2]. In all the cases, civilians were the main victims, it is estimated that between 1958 and 2012, the armed conflict in Colombia caused approximately 220,000 deaths, 37,094 kidnappings, and 80,514 forced disappearances [2]. According to the United Nations High Commission for Refugees, about 7.3 million people were internally displaced in Colombia from the beginning of the conflict through 2016, and approximately 50% of them were women. Which makes Colombia the second highest country in the world in terms of internal displacement, after Syria, this taking into account the contextual and temporal differences of the armed conflict in the two countries, with Syria having a decade of conflict and Colombia five decades [3].

At a global level, studies show that in a medium-sized country, conflict can increase malnutrition by 3.3%, reduce life expectancy by 1 year, increase infant mortality by up to 10%, and cause other public health problems due to lack of access to drinking water, food and health services [7]. Although the epidemiological profile and burden of disease of countries in conflict has changed in the last decades, infectious diseases like malaria, tuberculosis, leishmaniasis, cholera, acute diarrheal disease and poliomyelitis, continue to be serious problems [8–10]. Conflict can also have devastating effects on sexual and reproductive health, neonatal health, mental disorders and non-communicable diseases, such as cancer, hypertension and diabetes [11–13].

In relation to maternal and neonatal health, studies show that in situations of conflict there is an interruption of prenatal care services, which leads to the late detection of complications, such as preeclampsia and eclampsia [14]. In addition, given the difficulties of accessing sexual and reproductive health services, there are difficulties in the delivery of contraceptive methods [15], an increase in teenage pregnancies, sexually transmitted infections, gender violence, and child marriages [16]. Some studies also report moderately higher rates of maternal and infant mortality in countries in conflict compared to others with similar socioeconomic and demographic characteristics, without the existence of armed conflict [17–19]. A systematic review also showed consequences such as low birth weight, spontaneous abortion, fetal death, prematurity, and premature rupture of membranes [20].

Despite the long-term armed conflict, Colombia has shown considerable advances in both poverty reduction and in some public health indicators over the last few years. A United Nations Development Program report recognized that Colombia is one of the countries in Latin American that advanced the most towards achieving the Millennium Development Goals [21]. The goals for poverty reduction set for 2015 were met a year ahead of time. In 2014, the percentage of individuals living in poverty was estimated at 28.5%, while 8.1% of individuals lived in extreme poverty, which amounts to half of what was observed at the turn of the century. School attendance reached 100% as of the beginning of 2000, and women’s participation in politics and in the labor force at that time has shown great advances compared to the previous decade. With regard to health indicators, the child mortality rate was successfully reduced by half. Despite these successes, inequities between regions are persistent [22, 23]. The goal of 45 maternal deaths per 100,000 live births has not been reached yet in Colombia, and in some regions the maternal mortality ratio was 324 deaths per 100,000 live births in 2017, compared to the national average of 50.7 [24]. The evidence about the health outcome most affected by armed conflicts is not consistent. Studies show that armed conflicts increase the crude death rate, not only because of the increase in violent deaths but also because of the increase in other causes. In some cases, during periods of conflict stabilization, deaths from infectious diseases such as diarrhea can overcome violent deaths [25].

Although it is recognized that the effects of conflict among men and women are different, few differential evaluations have been conducted. A review of Percival et al., reported that the gender literature focuses on women’s health and more specifically on reproductive and maternal healt [26]. Due to their reproductive role, women are much more vulnerable and have a greater need for assistance in health services, which increases in conflict situations. Studies conducted in conflict scenarios have shown an increase in maternal mortality [19], low birth weight and prematurity [27].

Taking into account the evidence available internationally about the impact of armed conflict on Reproductive, Maternal, Newborn, Child and Adolescent Health and Nutrition (RMNCAHN) [19, 27, 28] and the limited information available in Colombia on the subject, we conducted a study to describe the RMNCAHN situation in the context of the conflict and post-peace treaty periods in Colombia, between the years 1998 and 2016, both from a qualitative and quantitative perspective. Furthermore, we examined the interventions related to sexual, reproductive and child health services delivered by the public health system, as well as non-governmental and United Nations organizations. This study is part of a multi-country project conducted by the BRANCH Consortium (Bridging Research & Action in Conflict Settings for the Health of Women & Children) [29], with the participation of 10 countries in conflict: Afghanistan, Pakistan, Syria, Yemen, Somalia, South Sudan, Mali, Nigeria, Democratic Republic of the Congo, and Colombia.

## Materials and methods

This paper presents a the findings of a mixed methods case study conducted in Colombia, which was one of ten case studies conducted with the common objective of describing the impact of conflict on reproductive, maternal and child health, and exploring the provision of RMNCAHN interventions in conflict settings and factors that influence its implementation. The quantitative data available at the national level included a range of reproductive, maternal and child health indicators and their relationship with the conflict in 32 departments and 1122 municipalities. An important aspect missing from the quantitative data, however, was specific data on the role of intervention. In order to better understand how the interventions were implemented and their influence in different conflict settings, we collected qualitative data through interviews and focus groups, to provide an important complement to the available quantitative data. Using both data sources enabled us to make a comparison between two municipalities in the country, one with low intensity of the conflict and another with high intensity.

We present the methods of the quantitative and qualitative component separately. In the results section both methods were combined to better describe the findings. First, we provide a general description of children’s and women’s health; second, a description of the contextual factors influencing their health: third, the interventions, programs and RMNCAHN indicators, fourth, health service delivery and, finally, we describe the post-treaty programs.

### Quantitative component

#### Study design and data sources

The quantitative component of this study involved secondary data analysis from sources available between the years 1998 and 2016. Data sources included a) the Information System for Social Welfare (SISPRO) provided by the Ministry of Health and Social Protection of Colombia [30], which contains health care delivery data, epidemiological information, and vital statistics, b) population projections from the National Administrative Department of Statistics (DANE) [31], c) the National Nutrition Survey of Colombia [32], d) the Immunization Expanded Program, and d) the national Victims Registry (RUV) [33].

#### Outcome variables

We chose sixteen RMNCAHN indicators from the Countdown to 2030 initiative [34] for which data from Colombia were available and reliable. These indicators are described in Table 2.

**Table 2:** Selected indicators and data sources

Name	Numerator	Denominator	Source
Maternal Mortality Ratio	Number of deaths of women while pregnant or within 42 days of termination of pregnancy, irrespective of the duration and the site of the pregnancy, from any cause related to or aggravated by the pregnancy or its management, but not from accidental or incidental causes.	Total number of live births	Vital Statistics (DANE) 1998–2004 SISPRO 2004–2016
% Antenatal care (four or more visits)	Number of women attended at least four times during pregnancy by any provider (skilled or unskilled) for reasons related to the pregnancy	Total number of women who had a live birth occurring in the same period	Vital Statistics (DANE) 1998–2004 SISPRO 2004–2016
% Skilled attendant at birth	Number of live births to women attended during delivery by skilled health personnel (doctor, nurse, midwife or auxiliary midwife)	Total number of women who had a live birth occurring in the same period	Vital Statistics (DANE) 1998–2004 SISPRO 2004–2016
% Institutional delivery	Number of deliveries occurring in a health facility	Total number of women who had a live birth occurring in the same period	Vital Statistics (DANE) 1998–2004 SISPRO 2004–2016
% Caesarean Section	Number of women delivering a baby by C-section	Total number of women who had a live birth occurring in the same period	Vital Statistics (DANE) 1998–2004 SISPRO 2004–2016
Body Mass Index in women (< 18.5 kg/m2)	Number of women 15–49 years old with BMI below 18.5 kg/m2 (not counting pregnant women)	Total number of 15–49 years old women (not counting pregnant women)	Encuesta Nacional de la Situación Nutricional en Colombia ENSIN-2005 - 2010
Neonatal Mortality Rate (0–27 days)	Number of deaths occurring during the first 27 days of life	Total number of live births	Vital Statistics (DANE) 1998–2004 SISPRO 2004–2016
Early Neonatal Mortality Rate (0–7 days)	Number of deaths occurring during the first 7 days of life	Total number of live births	Vital Statistics (DANE) 1998–2004 SISPRO 2004–2016
Infant Mortality Rate (< 1 year)	Number of deaths in the first year of life (0–11 months old)	Total number of live births	Vital Statistics (DANE) 1998–2004 SISPRO 2004–2016
% Measles, mumps, rubella immunization coverage	Number of children ages 12–23 months who are immunized against measles in a given year	Total number of children aged 12–23 months	Extended Vaccination Program (PAI)
% Poliomyelitis immunization coverage	Number of children ages 12–23 months who survived the first year receiving at least 3 doses of poliomyelitis vaccination	Total number of children aged 12–23 months	Extended Vaccination Program (PAI)
% Three doses of combined diphtheria/tetanus/ pertussis vaccine	Number of children ages 12–23 months receiving three doses of diphtheria/tetanus/pertussis vaccine (DTP3)	Total number of children aged 12–23 months	Extended Vaccination Program (PAI)
% Three doses of *Haemophilus influenzae* type B coverage	Number of children aged 12–23 months receiving three doses of *Haemophilus influenzae* type B vaccine (Hib3)	Total number of children aged 12–23 months	Extended Vaccination Program (PAI)
Age-Specific Fertility Rate (adolescents 10–19 years old)	Number of births to adolescents 10–19 years old	Total number of adolescents 10–19 years old	Vital Statistics (DANE) 1998–2004 SISPRO 2004–2016
Age-Specific Fertility Rate (adolescents 15–19 years old)	Number of births to adolescents 15–19 years old	Total number of adolescents 15–19 years old	Vital Statistics (DANE) 1998–2004 SISPRO 2004–2016
Fertility Rate	Number of births to women 15–49 years old	Total number of women 15–49 years old	Vital Statistics (DANE) 1998–2004 SISPRO 2004–2016

#### Exposure variable

In order to explore the association between the exposure to armed conflict and each of the outcome indicators, we calculated a variable that reflected the degree of intensity of the armed conflict. We used data from the RUV, in which all people considered to be victims of the armed conflict in Colombia are registered. The law in Colombia defines “victims” as individuals who have suffered from infractions of International Humanitarian Law or serious human rights violations that occurred during the internal armed conflict, but not because of common crime [35]. The data base records “victimizing incidents” in the context of the armed conflict, and identifies individual and collective violence episodes. At individual level, victimizing incidents include crimes against sexual integrity, recruitment of children and adolescents, homicide, torture, physical or psychological damage, forced disappearance, kidnapping, threats, and anti-personnel mines. At collective level, victimizing incidents include displacement, confining, loss of personal belongings or properties, land abandonment or dispossession of land, and terrorist acts.

Using the RUV database, we calculated a rate of victimizing incidents for each of the 1120 Colombian municipalities for every year between 1998 and 2016. The numerator was the number of victimizing incidents to women in x year for x municipality and the denominator the total number of women in the same year and municipality. Municipalities were divided into quintiles in relation to their victimization rates, the quintiles were used to analyze each indicator, comparing the highest quintile Q1 (municipalities with the highest rate of victimizing incidents) with the lowest quintiles Q5 (municipalities with the lowest rate of victimizing incidents). Additionally, in order to explore if violence episodes at the individual or collective level differentially affected health indicators, we calculated a rate of victimization using the steps previously explained, separately for those affected by individual-level victimizing incidents and another rate for the collective level. The indicators were subsequently averaged according to the milestones of the armed conflict declared by the literature (1998–2004, 2005–2011 and 2012–2016). Subsequently, the gap between the municipalities of quintile one and quintile five was analyzed for each indicator, that is, the group of municipalities that presented “very high” victimization vs. those with “low”.

The decision to calculate the victimization index only for women was mainly because 11 of the 16 indicators selected are directly related to women’s health and the interventions they should receive during pregnancy and birth. The remaining five, mortality in children under 1 year and four related to vaccination, depend largely on the actions taken by the caregiver, which in most cases in Colombia is the mother or a surrogate woman.

### Quantitative analysis

We constructed rates, ratios and proportions for each municipality and year, taking into account the numerators and denominators presented in Table 2. Those indicators by year and municipality were later grouped by conflict quintiles (exposure variable) and then we calculated summary measures for three periods of the armed conflict (1998–2004, 2005–2011 and 2012–2016) [36], meaning that for each of the three periods we had all the indicators for every one of the municipalities. In order to determine if the differences observed in indicators during each of the three periods between the highest and lowest quintiles were statistically significant, relative differences and their confidence intervals were compared using the Rothman and Greenland method [37]. Excel, SPSS version 23 [38] and R [39] were used for data analysis.

### Qualitative component

This component had the purpose to explore the relationship between the conflict and the delivery of interventions in sexual, reproductive and child health services, through the experiences and perspectives of the persons and organizations involved. Therefore, we included officials at the national and departmental (State) level, who provided an institutional perspective of the situation, specifically in the detailed description of the delivery, difficulties and needs. Personnel at the local level provided a local vision through concrete experiences, to understand the structural barriers and the direct impact of the delivery of interventions and health care during conflict.

A case study of two municipalities was undertaken. The selection of these municipalities was determined by the number of victims in each municipality, demographic similarities (comparable ethnic groups are present in both municipalities), accessibility, and safety conditions for the field team. The municipalities are located in the Department of Antioquia, which has the highest number of victims reported in Colombia [40]. One of the municipalities selected had high levels of conflict and is located in the western region of the department, and the other had low levels of conflict and is located in the southwest region.

#### Data collection and study participants

Data collection took place between June and August 2018 and was conducted by five trained researchers. Forty-one semi-structured interviews and eight focus groups were carried out, with 89 participants: 26 males and 63 females (Table 3). Interviews and group sessions were recorded on audio and transcribed for analysis. The BRANCH consortium provided four interview guides that were used in all countries included in the study, which covered issues such as interventions delivered and prioritized, contextual factors and changes that influenced the delivery of the interventions, and stakeholder coordination. The guides were specific for each type of informant: health personnel, government officials, and United Nations and NGOs personnel. In Colombia, a guide was created for non-institutional and academia key informants, covering the same issues.

**Table 3:** Number of interviews and focus groups

PLACE	Un agencies^**1**^	Government agencies^**2**^	Healthcare professionals^3^	Non-governmental organization	Key informants		Total people
					Academics	Community^4^	
**Medellín**	4	1	3	0	3	2	13
**Bogotá**	4	1	0	3	2	0	10
**Dabeiba**	0	2	9	0	0	2 4 FG: 21 people	34
**Jardín**	0	2	2 1 FG: 3 people	0	0	1 3 FG: 24 people	32
**TOTAL**	8	6	17	3	5	50	89

^1^ United Nations Development Programme, United Nations, Office of the United Nations High Commissioner for Refugees, International Organization for Migration, United Nations Population Fund, Panamerican Health Organization

^2^ Officials of the Ministry of Health, Secretary of Health of the Department of Antioquia, Local Secretaries of Health, and other officials of institutions related to sexual and reproductive health

^3^ Nurses, physicians, specialists, psychologists

^4^ Women’s associations, community organizations, indigenous organizations, victim’s organizations

FG: focus group

We conducted interviews in Medellín and Bogotá with officials from the Ministry of Health, departmental and local Health Departments, professors and researchers from universities, directors of national and international NGOs and representatives of the United Nations offices in Colombia. They were selected through intentional sampling, taking into account their experience in the subject of study and their availability to participate.

The participants who were interviewed in the two municipalities included community organizations and victims’ organizations delegates, indigenous communities’ leaders, health providers, FARC political party members, and municipal officials. These were selected tracking organizations or persons that had knowledge or had been involved in the armed conflict in the period of greater intensity and in the post-agreement, institutions responsible for the provision of programs and services to women, girls and boys were also included.

### Qualitative analysis

The qualitative information was analyzed based on pre-established dimensions used in all countries participating in the study. These dimensions included access and quality of care; interventions/programs; health care; health personnel; contextual problems and factors that can influence health delivery; and changes in the set of interventions implemented during the post-treaty period. The five researchers that conducted the interviews coded their transcripts, divided by geographical areas. The coding was done in Excel matrices designed by the researchers, according to the pre-established dimensions. Afterwards, a summary was made for each of the sites, and the qualitative information was collated in a single document.

## Results

### Health status of women and children

**Fig. 1:**
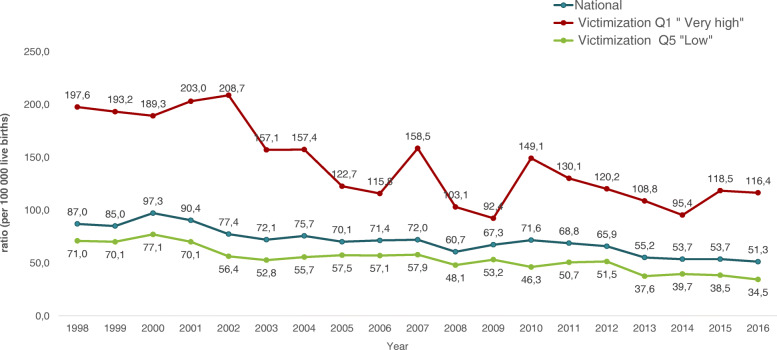
Maternal mortality ratio according to victimization quintiles. Colombia. 1998–2016

**Fig. 2:**
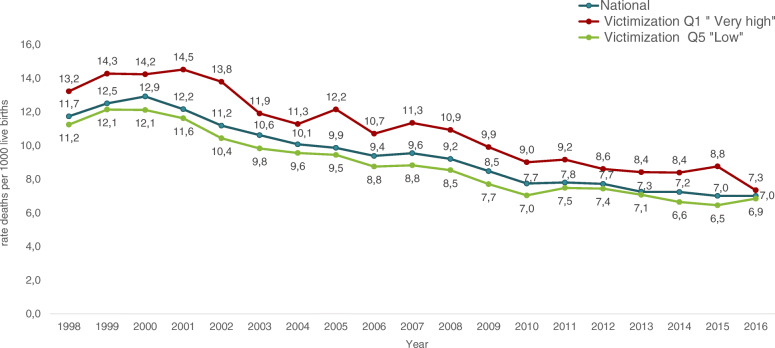
Neonatal mortality rate according to victimization quintiles. Colombia. 1998–2016

**Fig. 3:**
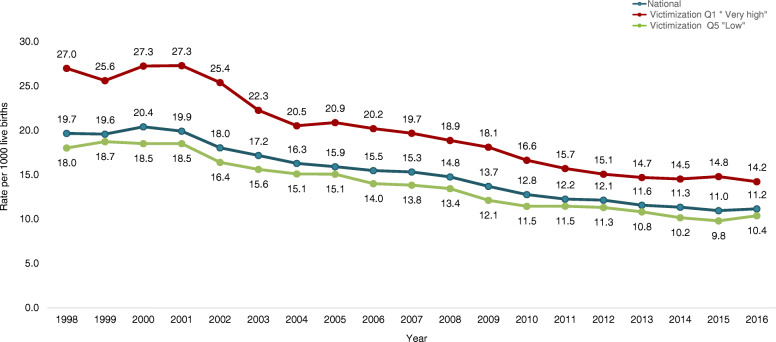
Infant mortality rate according to victimization quintiles. Colombia. 1998–2016

Based on quantitative analyses, RMNCAHN indicators in the country have improved considerably between 1998 and 2016. Table 4 shows the percentages of change in the rates of maternal mortality, fertility, neonatal and infant mortality, and malnutrition in women of childbearing age. Rates of maternal (Fig. 1), neonatal (Fig. 2) and infant mortality (Fig. 3) have decreased by about 40%, and fertility rates have also decreased by about 17 to 27% depending on the age of the woman. On the other hand, vaccination, antenatal care, institutional delivery and maternal health care by a skilled birth attendant have increased. For instance, percentages of vaccination -except for Haemophilus influenza type B (730%)- have increased by about 80%, and antenatal care has increased by 85%. However, when these indicators are analysed based on the levels of conflict (Table 5) the most unfavourable results are found in the municipalities with the highest levels of conflict, with statistically significant differences in maternal mortality, antenatal care, caesarean section, and adolescent fertility rate (15–19 years of age). Results will be presented and discussed in the following sections.

**Table 4:** Percentage of variation of RMNCAHN indicators in Colombia between 1998 and 2016

Indicator	1998	2016	Percentage of variation
Maternal Mortality Ratio	87.0	51.3	−41.0
Neonatal Mortality Rate	11.7	7.0	−40.2
Early Neonatal Mortality Rate	8.8	5.0	−43.2
Infant Mortality Rate (≤12 months)	19.7	11.2	−43.1
% of Antenatal Care (≥4 visits)	47.7	88.4	85.3
% Deliveries with skilled birth attendant	93.4	99.0	6.0
% Institutional deliveries	92.7	98.9	6.7
% Cesarean Sections	24.9	45.8	83.9
Fertility Rate (10–19 years of age)	39.3	32.3	−17.8
Fertility Rate (15–19 years of age)	79.1	61.6	−22.1
% Measles, mumps, rubella immunization coverage	51.5	92.6	79.8
% Poliomyelitis immunization coverage	51.1	91.2	78.5
% DPT3 immunization coverage	50.3	91.3	81.5
*% Haemophilus influenzae* B coverage	11.0	91.3	730.0

*RMNCAHN* Reproductive, Maternal, Newborn, Child and Adolescent Health and Nutrition

**Table 5:** Indicators by quintiles of violence with relative differences and confidence intervals according to time periods

Indicator	1998–2004					2005–2011					2012–2016				
	Indicator Q5	Indicator Q1	Relative differences	95% CI LL	95% CI UL	Indicator Q5	Indicator Q1	Relative differences	95% CI LL	95% CI UL	Indicator Q5	Indicator Q1	Relative differences	95% CI LL	95% CI UL
Maternal Mortality Ratio	65.0	185.8	**2.86**	**2.47**	**3.30**	53.1	124.1	**2.34**	**1.96**	**2.79**	40.4	111.8	**2.77**	**2.30**	**3.33**
Total Fertility Rate	63.8	62.6	0.98	0.77	1.26	54.9	62.4	1.14	0.89	1.46	50.0	56.8	1.14	0.88	1.47
Neonatal Mortality Rate	7.7	10.0	1.30	0.70	2.42	6.0	8.1	1.35	0.68	2.68	4.9	6.0	1.24	0.56	2.76
Early Neonatal Mortality Rate	11.0	13.3	1.21	0.70	2.06	8.3	10.5	1.27	0.69	2.32	6.9	8.3	1.21	0.61	2.38
Infant Mortality Rate (≤12 months)	17.3	25.0	1.44	0.97	2.13	13.1	18.6	1.42	0.90	2.24	10.5	14.7	1.40	0.84	2.33
% of Antenatal Care (≥4 visits)	66.5	43.0	**0.65**	**0.48**	**0.87**	86.5	72.4	0.84	0.67	1.05	89.5	77.9	0.87	0.70	1.09
% Deliveries with skilled birth attendant	98.5	85.2	0.87	0.70	1.07	99.4	94.2	0.95	0.77	1.16	99.7	95.7	0.96	0.79	1.17
% Institutional deliveries	98.2	83.1	0.85	0.68	1.05	99.4	94.3	0.95	0.78	1.16	99.7	95.6	0.96	0.78	1.17
% Cesarean Sections	32.9	15.7	**0.48**	**0.29**	**0.78**	40.3	29.2	0.72	0.50	1.04	47.3	39.3	0.83	0.61	1.14
Fertility Rate (10–19 years of age)	36.0	36.1	1.00	0.73	1.39	33.6	39.7	1.18	0.87	1.61	30.7	39.7	1.29	0.95	1.76
Fertility Rate (15–19 years of age)	70.0	75.3	1.08	0.86	1.35	65.3	79.2	1.21	0.97	1.51	58.4	76.5	**1.31**	**1.05**	**1.64**
% Measles immunization coverage	78.0	70.7	0.91	0.72	1.14	95.3	89.1	0.93	0.76	1.15	95.2	88.0	0.93	0.75	1.14
% Poliomyelitis immunization coverage	78.1	68.7	0.88	0.70	1.12	94.4	86.8	0.92	0.74	1.13	94.6	82.9	0.88	0.71	1.09
% DPT3 immunization coverage	75.7	73.2	0.97	0.77	1.22	94.4	87.0	0.92	0.75	1.14	94.8	83.1	0.88	0.71	1.09
*% Haemophilus influenzae* B coverage	59.7	50.4	0.84	0.64	1.11	95.0	86.0	0.91	0.73	1.12	94.7	83.0	0.88	0.71	1.09
% Women aged 15–49 with Body Mass Index < 18.5 kg/m2*						21.5	16.1	0.75	0.46	1.22	6.4	4.6	0.72	0.29	1.79

Indicator Q5: Indicator value for municipalities in quintile 5 (lower conflict)

Indicator Q1: Indicator value for municipalities in quintile 1 (higher conflict)

Relative difference: between quintile 5 and quintile 1

95%CI LL: 95% Confidence interval Lower limit. 95%CI UL: 95% Confidence interval Upper limit

Bold: Statistically significant differences

*Data available for 2005 and 2010 only

### Contextual factors that influenced RMNCAHN health care

Both municipalities studied as part of the qualitative component - particularly the municipality with higher conflict levels - exhibited contextual factors that exacerbated the effects of the armed conflict on the health of the population. Education levels in rural areas, geographical, communication and economic barriers, and cultural factors such as a *machismo*, in addition to problems within the health system, interfere with access to health services as mentioned by key informants. This was further aggravated by the prolonged presence of armed actors, who also targeted women and children.

*“Indeed, this rupture in the network of services, the fact that the State has not had a presence for so many years in those territories in conflict means that there is no guarantee of the rights of the population in general, so there is no education, there is no health, there is no housing, and there is no drinking water. Most of these sites do not have drinking water, they do not have sewerage, which is part of not having had State presence for five decades”.* United Nations official, Bogotá.

Disseminating information on sexual and reproductive health in remote communities was and still remains a challenge. In the municipality with higher conflict levels, for example, the indigenous leaders interviewed reported that the armed conflict exacerbated the circumstances leading to cases of maternal and neonatal mortality and child malnutrition. However, those are not officially recorded in all cases. A remark from a United Nations official in Bogotá also reflects this situation.

*“Still in Colombia there are confined populations. In Catatumbo, in Cauca, in Nariño, there are populations that cannot leave because of the conflict conditions. If I have a pregnant woman and I need to move her, she cannot leave, she can die, because there is no possibility of moving her. Because in any case women have been used in those territories as weapons of war and have been victims of violence”* United Nations official, Bogotá.

### Interventions, programs and indicators in RMNCAHN

In Colombia, interventions and programs related to RMNCAHN follow national and local guidelines and policies, which are delivered by health service providers hired by insurance companies covering the public and private health care regimes. Such interventions and programs were aimed at the general population nationwide. It has been only since 2011 that the government has offered health interventions throughout the country, designed specifically to provide victims of the armed conflict with comprehensive health and psychosocial care either individually or collectively. Additionally, over the last decades, interventions delivered by non-governmental organizations and United Nations agencies were reported in conflict zones, targeting displaced populations. NGOs have focused especially on mental health, gender-based violence, and provision of contraceptive methods. United Nations interventions varied, but have been mainly focused on human rights empowerment and humanitarian assistance. Below we present the integrated quantitative and qualitative results reflecting some of the achievements and challenges of the main programs related to RMNCAHN in Colombia.

#### Reproductive, maternal and newborn health

In the year 2000, the central government approved a guideline which outlined activities, procedures and interventions to increase demand for prevention and early detection of health problems, such as antenatal care, vaccination, growth and development assessment, adolescent health care, family planning and screening for cervical cancer. Although providing these services is mandatory both for private and governmental health insurance companies, the access to and quality of these services is not always optimal due to lack of qualified personnel, scarce availability or lack of supplies and medication, difficult geographic access, and lack of infrastructure and financial resources. Likewise, the armed conflict affected access to programs due to - among other things - difficulties in hiring health personnel, and the difficulties faced by health personnel in accessing the communities, and vice-versa. Moreover, there are no differentiated guidelines based on the socio-cultural and geographic context of each region, thus causing problems in implementation. The following quote illustrates the situation:

*…“It is not only the presence of armed conflict, but also the limitations in terms of equipment and medication needed to provide care in these zones that result in medical staff frequently leaving their posts after 6 months.”* Key academic informant, Bogotá.

As previously mentioned, the provision of antenatal care is compulsory for health insurance companies and local governments. In the year 2000, antenatal care guidelines were established to support the early detection of pregnancy disorders. Analysis of national statistics showed that the percentage of women receiving four or more antenatal care visits in the country increased from 48% in 1998 to 88% in 2016; these rates were lower in municipalities with higher victimization rates, with statistically significant relative differences for the period 1998–2004 (Table 5). This means that between 1998 and 2004 pregnant women in municipalities with higher victimization rates were 35% less likely to have antenatal care than in municipalities with lower victimization rates. There were no differences in antenatal care based on the rates of individual or collective victimizing incidents (Table 6). According to the qualitative findings, the lower antenatal care coverage in municipalities exposed to higher levels of conflict could be explained - to a certain extent - by restrictions in mobility, confinement, threats, checkpoints and armed encounters that prevented women from attending antenatal care services. Additionally, indigenous populations reported facing geographic and/or cultural barriers.

**Table 6:** Indicators by quintiles of individual and collective level victimizing incidents with relative differences and confidence intervals according to time periods

Indicator	1998–2004					2005–2011					2012–2016				
	Indicator Q5	Indicator Q1	Relative difference	95% CI LL	95% CI UL	Indicator Q5	Indicator Q1	Relative difference	95% CI LL	95% CI UL	Indicator Q5	Indicator Q1	Relative difference	95% CI LL	95% CI UL
Maternal Mortality Ratio (**ILVI**)	66.1	150.5	**2.28**	**1.94**	**2.67**	55.1	107.6	**1.95**	**1.62**	**2.36**	41.2	85.0	**2.06**	**1.67**	**2.55**
Maternal Mortality Ratio (**CLVI**)	64.9	185.4	**2.86**	**2.47**	**3.30**	53.8	124.6	**2.32**	**1.94**	**2.76**	40.4	112.5	**2.79**	**2.32**	**3.35**
% of Antenatal Care (≥4 visits) (**ILVI**)	66.2	45.7	**0.69**	**0.52**	**0.92**	86.2	73.9	0.86	0.68	1.08	89.2	79.7	0.89	0.72	1.11
% of Antenatal Care (≥4 visits) (**CLVI**)	65.8	43.1	**0.65**	**0.49**	**0.88**	86.4	72.4	0.84	0.67	1.05	89.6	77.9	0.87	0.70	1.09
% Cesarean Sections (**ILVI**)	33.1	15.1	**0.46**	**0.27**	**0.75**	40.7	26.7	0.66	0.45	0.96	47.6	35.4	0.74	0.53	1.03
% Cesarean Sections (**CLVI**)	32.3	15.7	**0.48**	**0.30**	**0.80**	39.7	29.2	0.74	0.51	1.06	46.5	39.4	0.85	0.62	1.16
Fertility Rate (15–19 years of age) (**ILVI**)	69.6	88.9	**1.28**	**1.04**	**1.57**	65.3	85.7	**1.31**	**1.06**	**1.62**	58.7	79.4	**1.35**	**1.09**	**1.69**
Fertility Rate (15–19 years of age) (**CLVI**)	71.1	75.4	1.06	0.85	1.33	65.8	79.4	1.21	0.97	1.50	58.6	76.7	**1.31**	**1.05**	**1.64**

(ILVI) Individual level victimizing incidents include crimes against sexual integrity. Recruitment of children and adolescents. Homicide. torture. Physical or psychological damage. Forced disappearance. Kidnapping. threats. and anti-personnel mines

(CLVI) Collective level victimizing incidents include displacement. Confining. loss of personal belongings or properties. Land abandonment or dispossession of land. and terrorist acts

Indicator Q5: Indicator value for municipalities in quintile 5 (lower conflict)

Indicator Q1: Indicator value for municipalities in quintile 1 (higher conflict)

Relative difference: between quintile 5 and quintile 1

95%CI LL: 95% Confidence interval Lower limit. 95%CI UL: 95% Confidence interval Upper limit

Bold: Statistically significant differences

Health institutions in Colombia can become certified as a Women and Child Friendly Institution (IAMI), a strategy promoted since 1990 by the national government together with UNICEF [41]. The purpose of this strategy is to guide, implement and evaluate actions to improve the health and nutrition of women, mothers and children, focussing on pregnancy, childbirth, the neonatal period, and growth and development of children up to 6 years. Although adopting this strategy was not compulsory, the hospital in the municipality with low levels of conflict, was certified as an IAMI institution in 2007. By contrast, this was not possible in the site with high levels of conflict, owing to difficulties in accessing econonomic resources and personnel, and the priorization of other activities relevant to a conflict zone. Currently, in the post-treaty period, more resources have been forthcoming to strengthen the institution, and the hospital in this municipality is now involved in the implementation of the strategy.

Regarding family planning, the national guidelines established in the year 2000 require that health care providers must implement intra and extra-mural educational activities, provide counseling and deliver contraceptive methods. However, qualitative findings reveal that this happens mainly in towns, and consequently rural communities have limited access to reproductive health education and contraceptive methods. Additionally, the variety of methods available in rural areas is limited, and cultural barriers like *machismo* and fear of hormonal methods hampers their use. In zones with high intensity of conflict, women had limited access to family planning due to the interruption of extra-mural activities, seizure of medicines and supplies, and mobility restrictions. In some of these areas, contraceptive methods have been provided by NGOs and UN programs.

*“When the conflict started here in ‘97, even after 2001-2002, no one went to a [health] brigade, [health personnel] did not go to any, and they were cancelled completely due to lack of security for hospital staff”.* Health personnel, municipality with high levels of conflict.

#### Child and adolescent health

In order to combat malnutrition, beginning in 1936, the national government established different kinds of school feeding programs at the national level. In addition, there were regional initiatives, such as that of the government of Antioquia, which created a Food and Nutrition Security Management office in 2003 that implemented and supervised nutrition recovery centers where dietary complements were provided to pregnant women and children [42]. Regrettably, the impact of these programs on child nutrition was not measured systematically, nor were specific adaptations made for indigenous populations, people in conflict areas, or rural population implemented. Corruption has also affected the proper functioning of these programs, and their ongoing continuity depends on the interests and priorities of the current government. During the conflict, malnutrition was one of the major concerns of institutional and community key informants in the municipality with higher conflict levels. Anti-personnel mines, forced displacement, confinement and restrictions on the passage of foods to certain zones, contributed to lower availability of food and reduction in the means of economic subsistence.

“*At [the time of the conflict] there were many children with malnutrition problems, of course mine did not, because even if it was plantain that I ground them in order to not let them become undernourished, but there were many children who were … clearly malnourished... ” “They were malnourished, they died, many children died because there was no food to give them”, “Yes, even old people were hungry, old people and children were the most affected, because as far as food is concerned, we could not work as we worked before*”. Community informants, municipality with high levels of conflict.

As far as vaccination is concerned, the country implemented an expanded immunization program (PAI) in 1979, which in 2018, included 11 vaccines. Quantitative and qualitative findings show high vaccination coverage, even in places where the armed conflict was most intense. National average coverage for scheduled vaccines such as polio, DPT and MMR rose from approximately 51% in 1998 to 91% for polio and DPT, and 93% for MMR. For *Haemophilus influenzae* type b, coverage went from 11 to 91% in the same period. Although there were differences in vaccination coverage of about 10% in favor of municipalities with lower conflict levels, the differences were not statistically significant in any period (Table 5). Vaccination has been offered within mural and extra-mural programs on a free basis for all children, and had a relatively stable resource allocation. Furthermore, some vaccinators in the two municipalities involved in the case study belonged to the local indigenous population and had held this position for decades and according to participants, probably for this reason, armed groups and communities allowed them to do their duty with few restrictions. Still, there are geographical barriers and some municipalities have to resort to medical air brigades, which are costly and scarce. In addition, in some places, the training and hiring of indigenous vaccinators was complicated and lacked continuity.

#### Other reproductive and maternal health indicators

Other indicators analyzed indirectly illustrate the impact (or lack thereof) of the previously mentioned programs and interventions (Table 5). For example, between 1998 and 2016, amidst a national decrease in the Maternal Mortality Ratio (MMR) of 41% (87.0/100,000 vs. 51.3/100,000), MMR was higher in the municipalities with higher victimization, with relative differences of 2.6 (95% CI: 2.47, 3.30) for the period 1998–2004, 2.34 (95% CI: 1.96, 2.79) for the period 2005–2011, and 2.77 (95% CI: 2.30, 3.33) for the period 2012–2016. When analyzing MMR by type of victimization, we found that collective episodes had a greater impact on mortality than individual ones (Table 6), and this is supported partially by qualitative findings which indicate that restrictions on mobility and confinement played an important role in the lack of antenatal care and higher rates of maternal morbidity and mortality. Additionally, in both municipalities, we found that women from remote areas with problems during pregnancy or delivery did not have timely access to specialized obstetric care. The capacity to successfully manage pregnancy complications and emergencies in the first level of care was poor, in part due to lack of skilled health personnel.

*“And we know that the causes of maternal death are more or less the same causes, which are basically hemorrhages, preeclampsia, and sepsis. These are the main causes of death and clearly have an origin in two actions that are complex to address, which are cultural barriers, geographical barriers and issues related to the quality of services.”* United nations official, Bogotá*.*

Cesarean section in all women with a live birth rose by 85%, increasing from 25% in 1998 to 46% in 2016. The proportion of women delivering by Cesarean section was lower in municipalities with higher victimization rates, with statistically significant differences only for the 1998–2004 period: relative difference 0.48 (95% CI: 0.29, 0.78). The increase in Cesarean section was also associated with high percentages of institutional births and deliveries with skilled birth attendants, which in 1998 were at 84 and 93%, and in 2016 at 98 and 99%, respectively. Differences in the percentage of institutional births between high and low violence municipalities were not statistically significant, and no differences were found according to the level of individual and collective victimizing incidents. On the other hand, between 1998 and 2016 neonatal, early neonatal and infant mortality rates fell by approximately 40%, with no significant differences between the high and low conflict quintiles.

Quantitative findings show that overall, the fertility rate in children and adolescents has decreased in the last two decades. Yet when the intensity of the conflict is taken into account, only the municipalities with low conflict level show a fall in fertility rates; rates rose in municipalities with higher conflict levels. The fertility rate in adolescents between 10 and 19 years old descended from 38/1000 women in 1998 to 27/1000 in 2016 in the lowest violence quintile, while in the highest quintile the rate rose from 32/1000 to 37/1000. For adolescents between 15 and 19 years old fertility rates dropped from 74/1000 to 52/1000 in the lowest quintile, and for those in the highest quintile of violence fertility rose from 68/1000 to 71/1000. Relative differences were statistically significant only for the fertility rate of 15 to 19-year-olds in the 2012–2016 period; municipalities in the quintile of higher victimization presented a rate of 76.5/1000 and those in the quintile of lower victimization 58.4/1000 (Relative difference: 1.31, 95% CI:1.05,1.64). However, when the type of victimization is considered in the analysis (Table 6), we found that for individual level victimizing incidents, the differences in fertility rates were significantly higher in municipalities with higher victimization rates (Q1) in all the periods 1998–2004: Q1: 88.9/1000 vs Q5: 69.6/1000, 2005–2011: Q1: 85.7/1000 vs Q5: 65.3/1000, 2012–2016: Q1: 79.4/1000 vs Q5:58.7/100. At the collective level, statistically significant differences were only found in the period 2012–2016: Q1:76.7/1000 vs. Q5:58.6/1000.

Even though in the qualitative findings there was not a direct mention about adolescent pregnancy, the difficulties in access to family planning that are illustrated in the following quote, may have also played a role.

“Suddenly as for the time they [women] stopped visiting, it seemed very sad to me that people could not go to the town, I was very committed to the promotion and prevention services, family planning, I was always the one in charge of family planning, and suddenly finding pregnant women who were so judicious, claiming the pills or sending someone for them, and that they get pregnant, because one talked to them, [and they said] I could not send someone, I could not go to the town”.(Health Official, Municipality with high levels of conflict).

### Health service delivery in the context of active conflict

In municipalities where armed conflict was most intense, it was particularly difficult to guarantee the permanence of health personnel, who were threatened, kidnapped, and murdered, while hospitals and health centers were seized.

*“Some health promoters received training and their work was greatly appreciated in this territory because they were able to attend births … I remember the late Marcos (not his real name) who attended births, and they all had this ability. But that ended when -during the conflict- two health promoters were killed.”* Community informant, Municipality with high levels of conflict.

The municipality with high levels of conflict in our case study was particularly affected by high rotation of health personnel, a situation that did not occur in the municipality with lower levels of conflict.

“When they killed the doctor at the health center, we were left without a doctor for four months because obviously nobody is going to want, even ads were published here in Medellin, in the School of Medicine, the manager called several contacts and people said, well if they just killed the doctor I am not going, no way, then I do believe that there was a difficulty or surely there are still in some parts of the country to get health personnel to go and work in areas where the conflict represents a real threat to life.”(Health professional, Medellín).

Because of the intensity of the armed conflict, extra-mural services that focused on the rural population were suspended in the municipality with higher levels of conflict for nearly 10 years. This affected delivery of sexual education and family planning programs, antenatal care, and child health programs. In 2005, as a result of the support of the International Red Cross Committee, extramural services were reactivated. Health personnel was trained on care protocols and on medical issues specific to armed conflict contexts, which helped to improve extramural services. On the other hand, extramural services were never suspended in the municipality with lower levels of conflict; instead, when armed groups where detected in rural zones, the community notified the health brigades who then postponed the outreach activities.

*“..I spoke on the issue of mobility restrictions, recently we were going to do an intervention with young people, on issues of sexual and reproductive rights and health, for reasons of the conflict in that area we could not, it’s recent, that was like four months or five months ago.”* United Nations Official, Medellin*.*

### Factors facilitating the delivery of health care

Informants from the municipalities reported that in cases where government officials and health care personnel belonged to the community, they were more deeply committed to their work and more willing to remain in service despite the difficulties, thus resulting in more stable services.

People in both indigenous and rural communities pointed out that their knowledge of herbs and traditional medicine allowed them to mitigate the effects of isolation and the barriers to access health care imposed by the conflict. Similarly, the existence of midwives, traditional healers, and health promoters in remote and rural settlements, along with the presence of NGOs, compensated to a certain extent for the effects of the armed conflict on the health of communities.

*“They [health personnel] didn’t come in or go out … the ones who lived out of town were afraid to come to town, and the ones from the town were afraid to go out into the country. Personnel from Doctors Without Borders came when someone was sick and they themselves took them away and gave them treatment.”* Community informant, municipality with high levels of conflict.

At times, collaboration among communities was the only option for saving lives in health emergencies. Actions like carrying a sick person by foot up to a health post, providing a vehicle or a telephone, or cultivating and exchanging products during times of food shortages, have played a crucial role in the health of communities. In rural areas, telephone services were limited or non-existent, and became even sparser during the armed conflict. Some communities used and still use telephones to communicate with health providers to make health decisions and receive instructions on attending emergencies.

Having strong communication with communities was critical to health service provision. Health personnel and officials from the United Nations report that a factor facilitating health care delivery was permanent contact with communities before entering the territories. Also, communication with leaders and a security analysis was important before starting any action in a community.

### Cooperation among stakeholders in the delivery of interventions

At government level, certain policies, strategies, and programs are adapted from guidelines of international organizations and UN agencies, such as the Pan American Health Organization. Prioritization of interventions by UN agencies in Colombia is based on indicators, surveys, situational analyses, and/or on direct requests from government offices. Populations most affected by the armed conflict are a priority for UN agencies. Additionally, UN officials mentioned that initiating actions in a territory and guaranteeing its operation requires institutional, cross-sectoral and inter-agency coordination. In most cases, non-governmental organizations and UN agencies work very closely at the national, provincial and local levels in order to achieve institutional strengthening and give continuity to some interventions. At national and provincial level, UN agencies work in thematic clusters, in which decisions are made on the issues to be prioritized, the interventions to be carried out and the way to proceed. Decision-making on types of interventions and beneficiaries, not only depends on these clusters, but also on mandates of the central and regional offices of agencies and donors.

According to some participants, donors sometimes determined where and how to execute strategies, a fact that limited the effectiveness of these interventions. Other difficulties encountered in continuing sexual and reproductive health programs included the scarcity of resources, lack of health personnel, or personnel without training in sexual and reproductive health, and weakness in the institutional capacity of private and public institutions. The following quote illustrates the lack of qualified personnel:

*“We have found that there are obvious shortcomings in the training of human talent, and it has everything to do with doctors and nurses, who do not know how to insert an IUD or to provide good antenatal care, nor how to handle maternal morbidity.”* United Nations official, Bogotá.

According to key informants, support from international agencies and national and international NGOs has been crucial to mitigate the effects of the armed conflict, and to achieve a long-lasting dialogue and a negotiated way out for the FARC-EP. Likewise, in the transition period, they have provided resources, training and support at a moment when the restoration of trust between the parties was paramount. For the communities themselves, such support has been relevant, albeit disjointed and intermittent.

In addition, a major player in supporting communities during the conflict has been the Catholic Church, as it has wide territorial presence. During the escalation of the conflict, it assisted the displaced populations and gave them shelter from armed confrontations.

### Post-treaty programs

After the signing of the peace agreement with the FARC-EP, a process of reincorporating its members into civilian life began in 24 Territorial Training and Reincorporation Spaces (ETCR, Spanish acronym), which were distributed across different regions of the country. These spaces were designed to facilitate re-entry of FARC-EP members to civilian life, develop projects aimed to generate economical resources and attend to the needs for technical training in the surrounding communities. Within the framework of the program, ex-combatants were affiliated with the Health and Social Security System. However, people interviewed reported some health care delivery problems because the insurance companies hired by the state do not have contracts with service providers throughout the country, a fact that obstructed access to health services for many ex-combatants.

Ex-combatants living in the ETCR had access to both general health care, and specific care regarding substance abuse and sexual and reproductive health such as gynecologist visits, access to sexual education activities, and contraceptives. The informants reported that in some instances health care interventions in the ETCR had no continuity, and also that there was reticence among ex-combatants to receive mental health care. Informants indicated that these situations still persist.

The government, together with NGOs and UN agencies, are implementing new sexual and reproductive health programs in the municipalities where the ETCRs are located. Nevertheless, many of these programs are generated at the central level, are applied without taking into account the context of the region and/or community, and with little participation of the community, therefore, health care delivery is considered deficient.

*It seems to me that in terms of sexual and reproductive health policies, there are policies that do not take into account the contexts, nor the cultures, nor the levels of affectation, nor the processes that are required, are processes, packages ... A flexible, adaptive policy is needed that starts by recognizing the conditions and needs initially and that in that perspective is adapted to the contexts and population groups. For example, in terms of sexual and reproductive health what is being done with the population in the process of reincorporation? I would say nothing, nothing different from having the services, having the possibility of a psychologist and they [ex-combatants] say that they do not need psychologists. But having the services does not mean that you have the option of the service, because one thing is to have the possibility of health and another thing is to be able to access health. How many of us are affiliated with the health system, but how many have difficulties in accessing, and if you access but you don’t have the medicines you don’t do anything either because you leave the office and you don’t have money, so I think there are many issues to review in the field of policies and on that subject too, I think it should be a closer process, more recognition. (*Key academic informant, Medellin).

*“Imagine that the hiring of PAPSIVI and our hiring were three months a year, that is, in one year I participated from October to December, the following year I participated from July to November and when I tell you, I mean the whole PAPSIVI at the national level, it worked that way … Then, those months, the population was totally neglected” (Health personnel, PAPSIVI).*

Moreover, there are redundant needs assessments and centralization of care in the most highly populated centers, as well as lack of interinstitutional and interagency coordination.

*Access to services is not the sole responsibility of the health sector, it is a road issue, infrastructure, technology, communication. If we want to improve health indicators, the health conditions of the population, and this is a perfect example of this, we are going to have a strong interagency strategy and that is also part of the National Rural Health Plan. If the quality of drinking water is very bad, because you can have all the doctors, but they will continue to get sick from the same gastric issues. If you want to solve the resolution capacity you can have all the telemedicine equipment but if you do not have effective communication, there comes MinTIC [Comunications Ministry] for example to play a very important role. The same in terms of access, the most frequent complaint in those areas, which I tell you PDT [territorial development plan] that are 170 prioritized municipalities is that the health service is far away, now, it is not so easy and not necessarily the answer is to build a health post. The answer may be if the road development were much better, for example, if you pave the road and you reduce the journey from four and a half hours to an hour and a half, believe me that you will substantially change the access, reduce costs and reduce time, are two things that are very important barriers for these areas in effective access. But that does not depend on health, we do not build roads, so I tell you that here the interagency issue weighs heavily in these remote areas. (Health official, Ministry of health).*

The final peace treaty to end conflict and build a stable and durable peace with FARC included the elaboration and implementation of a Rural Health National Plan. According to key informants, the formulation of the Plan took into consideration the social determinants of health, and highlighted differential approaches for women and children. The Plan is basically part of a wider commitment to deliver solutions to the issues already identified in different national policies. However, at the time of data collection, informants pointed out that it had not been implemented yet.

Regardless of the existence of public policies for comprehensive and inter-sectorial health care for victims, ex-combatants and inhabitants of the townships most affected by armed conflict, the policies were not applied automatically and required state- and national-level action to guarantee their implementation, since local governments did not always count on or allocate enough resources for their implementation, this issue persists.

Additionally, under the coordination of the Ministry of Health and in collaboration with the Multi-Donor Fund for peace, the International Organization for Migration, the Pan American Health Organization, and the United Nations Population Fund, the government provided training to ex-combatants and the population from areas surrounding the ETCRs to train them as health promoters to work with the rural population. Unfortunately, to date, very few of them have found a secure post in the health workforce, partly because the government did not facilitate their insertion into the labor market nor guarantee their labor security.

*“The government promised to fulfill from Havana [Peace treaty], things that are not being fulfilled, that we only hope that the homologation of knowledge will be given for us to be able to continue with our profession that was empirically done before and what we want is to professionalize and to have access one day to a position and to hold a position and to perform what one has done for so many years, but has not happened” (Nurse, FARC).*

Finally, it is important to mention that most of the actors interviewed recognized that the post agreement period did not bring about great changes in the delivery of interventions, or in health care in general. However, they mentioned the fact that it has been possible to re-enter to areas where the FARC previously had presence, and the population has then received extramural attention.

*“For me it is difficult to find a change due to the signature [of the peace treaty], if we start talking about the actors and the nature of the conflict itself, of the combats, they are a different issue, but if we are talking about health, in the projects we have, there has been no change. On the other hand, if we look at our statistics, we have much more [health care] attentions now.”* NGO Officer, Bogotá.

## Discussion

Despite the armed conflict lasting more than five decades, the results of this study highlight the progress made over the last two decades with regard to reproductive, maternal and neonatal health. However, maternal mortality and fertility in adolescents 15 to 19 years of age were statistically higher, while rates of Cesarean section and antenatal care were lower, in municipalities with higher levels of conflict as opposed to municipalities with lower levels of conflict. In Colombia, the health of women, children, and adolescents was affected by an interaction of factors, such as socioeconomic conditions, structure of the health care system, and limited availability of interventions, which were all exacerbated by the conflict. The national government mostly provided programs aimed at women and children, with difficulties in continuity and quality; these programs did not take into account the characteristics of municipalities with high levels of violence or those with ethnic populations. Among the barriers identified in the implementation of reproductive and child health interventions were the lack of supplies and health personnel, geographical barriers and insecurity, marked among others by mobility restrictions, confinement, food and medical supplies blockages and siege. The UN Agencies and NGOs had greater legitimacy in remote geographical areas that experienced intense armed conflict, where they carry out some health interventions.

There are great social inequities in Colombia which particularly affect the rural population and which worsened during the armed conflict. In this respect, it is important to recognize that the damages resulting from the armed conflict converged with high levels of poverty, lack of access to basic sanitation, unequal land distribution, and gender and ethnicity barriers. Additionally, it is important to take into account that in 1993, Colombia underwent a health system reform, where the state delegated a significant part of its function as guarantor of health rights to private and public insurance companies responsible for collecting and managing health The reform did increase health coverage, but due to multiple problems widely reported in the literature [34, 40, 43, 44], it did not guarantee universal access to quality services [45]. In spite of this, the Colombian government managed to offer a minimum set of health interventions to almost all of the municipalities in the country through a network of health centers and primary level hospitals [46].

Although the Colombian conflict has lasted over 50 years, only in 2011 did the national government issue specific legislation to regulate and provide guidance for care to victims of the conflict. However, according to Mogollón and Vásquez, this legislation is weak as it does not define the funding sources which will support the policies towards displaced population, therefore limiting its implementation [47].

Furthermore, Ramírez, Veloza and López concluded in their study that the response of the Colombian State has been insufficient in guaranteeing the right to health of the conflict’s victims. Through legislation and political discourse the State recognizes the responsibility of damage repair to these people but in practice this has not taken place [48]. By 2018, the victims had not benefited yet from a comprehensive and differential attention [48].

In our quantitative analysis, indicators related to neonatal or child health did not present significant differences. However, indicators related to pregnancy, showed statistically significant differences. This is probably related to the fact that antenatal care can be delivered by health personnel with basic training, while resolution of obstetric emergencies requires both highly trained personnel and effective referral systems. It is important to note that while the rates of Cesarean section in the non-conflict zones are much higher than those typically suggested by WHO, they are similar to other countries in the Latin and South America [49]. As such, they may indicate that facilities in non-conflict areas were more able to adhere to a standard of care that is consistent with regional medical norms, even if the rates are considered high by an international standard, than were facilities in the conflict areas.

While the contribution of access to care on health indicators’ levels was not assessed quantitatively, the results from the qualitative analysis suggest that barriers in access played an important role. Global evidence and particularly evidence in Colombia shows that access to health services for pregnant women is crucial to achieve adequate health outcomes [28, 50], and that this is even more critical in conflict areas, where access to emergency obstetric care is essential [51, 52]. On the other hand, low socio-economic level has also been reported in Colombia as a factor associated with poor perinatal outcomes [28], and as mentioned before, the population exposed to conflict also belonged to low socio-economic strata. It is a paradox, however, that according to quantitative findings, institutional delivery and skilled birth attendance, indicators that imply access to care, did not show significant differences between municipalities with high and low conflict levels. A possible explanation for this is that the quality of these services in municipalities with high conflict levels is poor and that -as found in our research- health personnel at the primary care level are unable to handle childbirth complications adequately, partly due to poor training, which at the end impacted maternal mortality.

Conflict is often associated with harmful shifts in social norms that promote violence, sexual abuse and macho culture. High adolescent fertility rates have been reported in other countries affected by conflict. Such is the case of Iraq, where adolescent fertility rate increased by 30% during the first year of the war, probably due to an increased prevalence of early marriage particularly among less educated women [53]. Likewise, a report from the National Health Institute of Colombia showed that the adolescent fertility rate increased among populations most exposed to the conflict [54]. The increased fertility rate among adolescent girls exposed to conflict in Colombia may be partially attributed to constraints in accessing services, such as disruptions in service and commodity availability, as well as increases in sexual violence, both of which may disproportionately affect young women. As such, programs should ensure adequate and continuous access to contraception and sexuality education should counteract shifts in gender norms, especially in areas heavily affected by conflict.

With respect to immunization, vaccination rates have declined in countries like Syria as a result of the conflict [55]. However, this is not the case in Colombia due to several factors related to implementation that have also been observed in other conflict settings. In our study, we did not find any statistically significant difference or qualitative evidence in vaccination coverage between municipalities that were more or less exposed to the conflict. Results from a study in Nigeria, Somalia and Pakistan found, as ours did, that the presence of permanent vaccination teams with personnel from local communities was an effective strategy to guarantee adequate coverage [56]. In addition, in Colombia a relatively stable resource allocation further contributed to the success of the program.

Organizational capabilities and knowledge of health issues by community health providers (i.e., traditional healers, birth attendants, community health workers, etc.) were crucial in alleviating the effects of the armed conflict, especially in more remote areas. The presence of midwives, traditional healers and health promoters in rural areas may have contributed to save lives. In cases in which institutional care cannot be guaranteed, traditional medicine should be valued and optimized by training, since in remote areas it may be the sole option for dealing with emergencies, poor transportation and lack of personal and institutional resources. Furthermore, our study as well as Franco’s [57], also undertaken in Colombia, found that health personnel lacked knowledge about human rights and international humanitarian law. Our findings revealed that in places where relevant training took place, there was improvement in the “Medical Mission” (*Misión Médica* in Spanish), enabling people to recognize situations of individual vulnerability and implement strategies to reduce lethal actions against health personnel [58]. Therefore, reinforcing this subject with health personnel in conflict areas seems to be very important, and NGOs and UN agencies have a key role to play in this type of training.

Colombia now faces great challenges in peacebuilding and in implementing policies for the transition from conflict to peace and the incorporation of guerrilla fighters into society. One of the characteristics of policies and programs emerging from the peace treaty signed with FARC that may have a positive impact on the health situation in Colombia is that they are not aimed exclusively at direct victims of the conflict and ex-combatants. For instance, the Ministry of Health has transferred resources to public hospitals located in the areas of the ETCR with the purpose of granting healthcare to population living in the area, including ex-combatants, key target populations of this effort are children and pregnant women. Likewise, with support of the UN Post-Conflict Fund, the project Health for Peace seeks to strengthen local capacities to improve access to comprehensive Primary Health Care services, with an emphasis on sexual and reproductive health, mental health, prevention of the consumption of psychoactive substances, child health and nutrition. The initiative is being implemented in 25 municipalities, where Development Programs with a Territorial Approach (PDET) were formulated and 24 ETCR had been established. In the treaty, under the category of Territorial Peace, the need to settle historical debts in regions where the persistence of armed conflict limited and discouraged institutional presence and action is recognized. It is important to mention that although the participants stated in the interviews that they did not perceive many changes, the execution of these programs had only started 1 year before the time when the study was carried out.

Finally, availability of health information for decision making is crucial in the allocation of resources and prioritization of interventions and programs. Although health information systems have advanced considerably in the last decades, obtaining updated data on sexual and reproductive health in remote communities is still a challenge.

Our study shows strengths and weaknesses worth mentioning. The main strength identified in the quantitative component is that the country has good quality national data, which come from vital statistics and epidemiological surveillance reports with low rates of under-reporting for public health events. This allowed carrying out national analyses and showing a complete picture of the indicators in relation to the armed conflict. A limitation is that the rates and ratios presented in the quantitative component were not age-standardized or smoothed. Additionally, we did not control for confounding variables such as poverty and education, and therefore it is impossible to fully disentangle the effect of the social or economic environment on the outcomes of interest in this paper. However, the qualitative data in this study help to provide additional support for there being a relationship between the experience of violence and the study outcomes and lend credibility to this study’s results. At the qualitative level, we highlight as a strength, the comparison between a municipality of high conflict versus a municipality of low conflict, which allowed us to isolate the potential contribution of the conflict, while recognizing that other problems of the country also played an important role. We also highlight the diversity of perspectives that were integrated into the work. In terms of limitations, the fact that the qualitative component only included two municipalities limits the generalizability of the study. Since the dynamics of the regions, the populations, the armed groups, and the level of conflict are diverse, generalizing to other municipalities or regions would not be appropriate. Another limitation was the inability of some respondents to reveal information considered by some organizations sensitive and/or confidential.

## Conclusions and recommendations

An important difference between the Colombian armed conflict and conflicts in other countries is that it was not a “total war” (mobilizes all of the resources of society to fight the war) For this reason, there was a certain degree of institutional stability despite the ongoing conflict, and health services were provided without interruption in some areas, especially in big towns and cities. Nonetheless, this increased the disparities in health status between rural and urban areas, as rural areas typically had a higher presence of armed actors, and many programs ceased in these regions resulting in the suspension of health services for prolonged periods. In these cases, attending to health care needs involved long trips under conditions of restricted and insecure mobility. Territorial differences in the effects of the conflict must therefore be taken into account when developing health protocols, programs and public health policies.

Sexual and reproductive health programs and interventions in conflict and post-conflict settings should be contextualized based on deep understanding of the underlying complexities of the situation. This involves building collective consensus among local stakeholders from the bottom up, instead of imposing actions based on policies that do not take local circumstances into account. It is a challenge that involves breaking down hierarchies and establishing a dialogue between professionals who bring their technical knowledge and communities with their own knowledge and beliefs. At the same time, institutional and technical capabilities need to be improved at municipal level; community health management strengthened, and the value of midwives, health promoters and traditional healers reassessed. We should aim for a better coordinated action between communities, institutions and multisector stakeholders.

Although, many programs are designed with a differential approach and the regulations that create them make this claim evident, they still cannot be executed in such a way that they meet the needs of the population. It would be necessary to do an investigation on this subject to identify what are the obstacles that prevent the proper implementation of the differential approaches, although this was not the object of our investigation, we consider that there are methodologies that allow the participation of different actors for the taking of decisions that could be implemented [59]. Although we do not mean that the central intervention is bad per se, dialogues with local and community actors, and in many cases autonomy with adequate oversight, are necessary and productive. Central accompaniment is essential, but it is also necessary to build capacities in the territories through trained professionals and adequate resources to propose solutions that are relevant and sustainable over time.

While this is a broad issue that has already been addressed, it deserves more attention, especially concerning the health of women, children and sexual and reproductive health [60]. The approaches with which this phenomenon has been analyzed are generally focused on mental health and leave aside other vital aspects such as health infrastructure, health personnel and specific effects on the population with differential approaches [61]. In addition, it is necessary for future studies to constantly monitor changes in health effects with the transformation of the armed conflict and the disappearance of an armed actor and the rearmament of others; as well as evaluate the implementation of what has been agreed in the National Rural Health Plan, to generate alerts on time and propose recommendations. Regarding mental health, it would be interesting that new studies can investigate the assessment and treatment of trauma in indigenous communities, where the perception of pain, disease and healing involve emotional and cultural aspects. In addition, mental health needs to be focused on victims, but also on ex-combatants and health personnel.

In turn, new studies could investigate the sexual and reproductive health of ex-combatants before and after the peace process in order to explore the changes and requirements of these women, and in men, propose sexual and reproductive health schemes with Gender approach. Finally, an analysis of the health programs and the contributions of the peace process to these programs can contribute to the understanding of the importance of peace for the consolidation of health institutions, staff security, and the consolidation of infrastructure, which in turn generates better indicators and strengthens public health.

## Data Availability

The data generated during and/or analyzed during the current study are not publicly available due to confidentiality of participants, but some of the information could available from the corresponding author on reasonable request.
